# Perspectives and Attitudes of Patients with COVID-19 toward a Telerehabilitation Programme: A Qualitative Study

**DOI:** 10.3390/ijerph18157845

**Published:** 2021-07-24

**Authors:** Carlos Bernal-Utrera, Ernesto Anarte-Lazo, Elena De-La-Barrera-Aranda, Laura Fernandez-Bueno, Manuel Saavedra-Hernandez, Juan Jose Gonzalez-Gerez, Maria Angeles Serrera-Figallo, Cleofas Rodriguez-Blanco

**Affiliations:** 1 Fisiosur I + D Research Institute, Garrucha, 04630 Almería, Spain; cbutrera@us.es (C.B.-U.); fisioelenacordoba@gmail.com (E.D.-L.-B.-A.); clinicasaavedra@yahoo.es (M.S.-H.); cleofas@us.es (C.R.-B.); 2 Physiotherapy Department, Faculty of Nursing, Physiotherapy and Podiatry, University of Seville, 41009 Seville, Spain; 3 Doctoral Program in Health Sciences, University of Seville, 41009 Seville, Spain; anartelazo.ernesto@gmail.com; 4 Department of Morphological and Socio-Health Sciences, University of Cordoba, 14071 Cordoba, Spain; 5 University Hospital Virgen del Rocio, 41013 Seville, Spain; laurafernandez0895@gmail.com; 6 Department Nursing, Physiotherapy and Medicine, Faculty of Almería, 04120 Almería, Spain; 7 Stomatology Department, Faculty of Dentistry, University of Seville, 41009 Seville, Spain; maserrera@us.es

**Keywords:** COVID-19, physiotherapy, telerehabilitation, exercise therapy, qualitative analysis

## Abstract

The total isolation of patients with coronavirus disease 2019 (COVID-19) requires non-face-to-face medical assistance. There is evidence of the efficacy of home treatments with exercises in patients with respiratory disorders which could become the therapeutic method of choice for the treatment and supervision of patients isolated due to infection during home confinement. This study’s objective was to analyse the experience and opinions of isolated patients with COVID-19 included in a programme of telerehabilitation exercises for 14 days and it is intended to reflect, from a qualitative point of view, the viability and usefulness of telerehabilitation tools in the management of these patients. Twenty-five participants of a telerehabilitation programme were interviewed by telephone through semi-structured interviews, following a positivist and objective model. The data were categorised and analysed through NVIVO qualitative analysis software. The information obtained was classified into four main topics (telerehabilitation programme, perception of clinical benefit, psychological aspects and level of health care) and six subtopics (technical aspects, communication, improvement aspects, exercise plan, motivation and applicability to public health systems). The telerehabilitation programme established in patients confined by COVID-19 is very well received, without considerable technical difficulties and generates physical and psychological improvements. Patients highlight the importance of applying this type of programme in public health systems.

## 1. Introduction

Since the first case of coronavirus disease 2019 (COVID-19) was confirmed in Wuhan, China, the virus dramatically spread and a pandemic was declared in March 2020 [1], with more than 120 million cases one year later [2] and leading to undesirable physical and psychological consequences to both healthcare providers and patients. Indeed, this situation has been defined as “an apocalyptic and unexpected war” [3,4]. Nonetheless, some authors argue that severe acute respiratory syndrom coronavirus 2 (SARS-COV-2) infection is the consequence of a systemic disease based on overpopulation, globalisation, hyperconnectivity and extreme centralisation and increasing fragility of supply chains [5].

Different strategies have been established all over the world to face this situation, with main measures that are based on movement control, coordination with surrounding countries and early detection of the virus by the performance of tests [6]. Closing/opening models have been shown to be the least expensive [7]. Other complex systems of radiodiagnostics, which are accessible, efficient and fast, have been developed, although they have not been implemented by all health systems [8,9].

One of the most common measures to mitigate the virus spread, however, has been isolation [6]. Isolation after being diagnosed with COVID-19 or being in contact with someone infected is one of the essential measures proposed by authorities. In that sense, the World Health Organisation (WHO) recommends for 80% of patients who do not require hospital admission the need for very restrictive home isolation and confinement in individual rooms at home to avoid dissemination of the virus [10]. However, home isolation limits activity and implies a notable deconditioning at the musculoskeletal level, indicating negative metabolic changes [11,12], repercussions on one’s emotional state and mental health decline [4,13,14].

In such a situation, a patient’s total isolation requires non-face-to-face medical attendance, using telematic control to monitor the evaluation of the patient affected by COVID-19. There exists evidence regarding the efficacy of domiciliary exercise-based treatments in patients with respiratory disorders. Based on this, it could become the therapeutic method of choice to allow the treatment and supervision of isolated patients due to infection during home confinement [15,16,17]. It has been suggested that a session of exercise through a telerehabilitation system may be a feasible option to manage quarantined patients with COVID-19 [18,19], enabling physiotherapists to provide interactive treatments by means of different devices [20]. 

Telemedicine and, more specifically, telerehabilitation have been analysed and recognised as feasible and practical options for managing different conditions and is an emergent area among healthcare providers [21,22,23,24,25]. However, the exceptional characteristics of COVID-19 patients, due to the isolation and saturation of public health systems [4], could become barriers to implementing and maintaining the implementation and maintenance of these methods in managing these patients. 

Therefore, this study’s objective was to assess and analyse the experiences and opinions of isolated patients with COVID-19 included in a telerehabilitation exercise-based programme for 14 days [19]. Our aim was to reflect, from a qualitative point of view, the feasibility and utility of telerehabilitation tools in managing these patients.

## 2. Material and Methods

### 2.1. Research Design

A qualitative exploratory study was conducted based on an interpretive framework following the Standards for Reporting Qualitative Research (SRQR) and Consolidated Criteria for Reporting Qualitative Research (https://www.equator-network.org/ accessed on 4 July 2021). The aim of this explorative descriptive qualitative study is to evaluate the participants’ viewpoints of this novel method of telerehabilitation, with the peculiarities of the SARS CoV-2 infection.

This research used the qualitative method through the semi-structured interviews of the patients affected by home confinement due to COVID-19 in a two-week telerehabilitation programme based on tonic and respiratory exercises [19]. Due to logistical aspects, we were obligated to reduce the planned intervention from the initial three to two weeks, primarily since 14 days was the time period that patients diagnosed with COVID-19 were obligated to stay at home in Spain. Interviews were performed in subjects who performed either the tonic or respiratory exercise programme.

### 2.2. Intervention

The telerehabilitation programme was based on two video calls at baseline and another during the middle of the programme, on day 7. During these video calls, physical therapists taught, revised and adapted exercises relevant to the patients’ perceived fatigue. In addition, a link to a website where exercises could be consulted was provided to the patients. Follow-ups were performed daily through WhatsApp; the objective of the follow-up was to ask the patients regarding any difficulty or trouble they might be experiencing with the exercises and any worsening health status. Participants were also given a telephone number that could be called in case of urgent contact.

### 2.3. Study Subjects

Using a random sampling method, we selected 25 of the 98 subjects studied in the telerehabilitation project from November 2020–January 2021. None of the respondents refused the interviews. Participants enrolled in the telerehabilitation programme presented with mild to moderate symptomatology due to COVID-19. They did not require hospitalisation and were confined in their homes after being tested positive by a polymerase chain reaction (PCR) test. The interviewed patients were required to meet the following criteria: (1) allowed the study of their speech regarding the interview and (2) completed at least 90% of the sessions of the telerehabilitation programme.

### 2.4. Interview Outline

We determined the interview’s scheme by consulting the relevant literature, seeking the opinions of those responsible for the telerehabilitation programme and covering all the areas of interest under our criteria. The main interview questions posed to the participants are listed in Appendix A. These questions were selected through a blinded poll by the seven members of the research group, who chose questions among a broad group of questions under their criteria and generated a semi-structured and complete interview. All the selected questions obtained at least five of seven votes.

### 2.5. Data Collection

One interviewer, with a Master’s Degree in New Healthcare Trends in Health Sciences, and the second interviewer, with a Master’s Degree specialised in Research Methodology, were previously coordinated and trained to interview for the purpose under study.

The individual interviews were scheduled at the interviewer’s convenience and carried out by telephone. The interviews were recorded and kept strictly confidential, with no one but the participants and researchers present during the sessions. The interviews lasted between 10–30 min for each participant. Study subjects were allowed to withdraw consent at any time. The researchers remained neutral in data collection and established good relationships with the participants, using active listening and clarification techniques to promote the authenticity of the data and avoid bias. Interviews were not repeated.

### 2.6. Data Analysis

The interviews were transcribed and analysed by NVIVO qualitative software. Two researchers independently reviewed the interview materials, summarised and extracted meaningful statements and formulated the presenting themes. Conflicting opinions on the contents of a theme were discussed and resolved by the director of the study. A thematic, inductive analysis consisting of a codification process from the participants’ narratives was performed. The information was divided into main themes and subthemes through codes.

### 2.7. Rigor and Trustworthiness

To establish trustworthiness of the data by reviewing the issues concerning data credibility, transferability, dependability and confirmability, specific strategies were used. Firstly, investigator triangulation, in which each interview was analysed by two researchers, occurred. Thereafter, team meetings were conducted, in which the analyses were compared and categories and themes were identified. Triangulation of the methods of data collection also occurred, in which semi-structured interviews were conducted and researcher field notes were kept. Researcher reflexivity was encouraged through previous positioning, performance of reflexive reports and description of the rationale behind the study. In-depth descriptions of the study were made, providing details of the characteristics of researchers, participants, contexts, sampling strategies and data collection and analysis. An audit by an external researcher was performed, in which an external researcher assessed the study research protocol, focusing on the aspects concerning the applied methods and study design. In addition, the external researcher specifically checked the description of the coding tree, the major themes, participant quotations, quotation identification and theme descriptions.

### 2.8. Ethical Review

This study complies with the Helsinki guidelines for human research and was approved by the ethics committee of the University Hospital Virgen Macarena -Virgen Rocio (ethics code: 1465-N-20). All participants signed informed consent forms.

## 3. Results

This study analysed 25 interviews regarding a telerehabilitation programme in patients with COVID-19 [19]. Participants did not provide feedback on the findings. Participants’ baseline characteristics are presented in Table 1.

The data analysis revealed 1568 codes and nine categories. In addition, four main themes (telerehabilitation programme, perception of clinical benefit, psychological aspects and health assistance level) and six subthemes (technical aspects, communication, aspects of improvement, exercise learning, motivation and applicability to public health systems) emerged from the data analysis.

### 3.1. Telerehabilitation Program

Participants were generally satisfied that they had participated in this telerehabilitation programme. They considered it conducive for them because they felt they took part in their treatment and recovery, which was a significant issue for them due to isolation. They appreciated the personalised care and follow-up performed by healthcare providers in uncertain moments of difficulty. It is noteworthy that very few of the interviewed participants were aware of the applicability of physical therapy in respiratory disorders. After completing the programme, all participants stated they would participate again if necessary and would agree to perform a telerehabilitation programme in other areas. Significant aspects highlighted by participants are detailed below and most highlighted answers can be found in Appendix B.

### 3.2. Technical Aspects

Regarding the technical aspects, the participants were not expected to encounter trouble performing the exercises since they were familiar with the instruments used. Most of the participants did not have any incidence concerning the technical aspects and a few participants had difficulties that they could solve quickly. Only one participant found incidences that could not be resolved and made no possible intervention to remedy them.

### 3.3. Communication

The contact form used during the video calls and messaging apps were qualified as well suited, primarily due to the circumstances of the pandemic. This communication was considered very useful and well adapted. Regarding the interaction, the possibility of being in daily contact with a therapist has been excellent. The level of confidence between the patient and therapist increased as the programme advanced. Participants referred to the professionalism of the therapist and they felt security and tranquility.

### 3.4. Exercise Planning

The learning and performance of the exercises were not challenging for the patients since they were easily performed through video calls. Small doubts were successfully solved by the physical therapists involved in the study.

Occasionally, symptomatology caused by COVID-19 made the programme’s development difficult; however, complications were solved by adaptations in the exercise programme in all cases.

### 3.5. Aspects to Improve

A minority of interviewed participants proposed options to improve our programme. They suggested that more information could be provided concerning the role of exercises for the body, what does they produce and why. Some considered that the programme allowed them to better understand how exercise works and how it can help in their recovery. The number of exercises and intensity were increased in those patients whose symptomatology subsided in a few days. Moreover, the time of the programme was also increased because when the participants completed the original 14-day regimen, they stopped performing the exercises, without the therapist’s follow-up or direction. Since it was considered that some patients still needed to continue with this intervention, the duration of the exercise programme was individualized.

On a technical level, pulsations were suggested for measurement of contrast sensations, as well as ways through the media for the older population to facilitate access to the programme. Nonetheless, despite these opinions, the programme appeared to have been innovative and easy to perform, a view shared by many participants who thought improvements are not needed.

### 3.6. Perception of Clinical Benefit

Regarding clinical benefit, most of the interviewed participants found positive evolution in respiratory capacity. In terms of physical symptomatology, such as fatigue, weakness and myalgia, they felt that they improved significantly since the exercises helped them drastically reduce symptoms. In addition, most patients did not only find improvement in the physical aspect, but also psychologically. Finally, a few participants did not know how to explain the clinical benefit since they experienced very mild symptoms.

## 4. Psychological Aspects

Concerning psychological aspects, most of the interviewed participants stated that the programme gave them emotional support because it became a distracting escape from confinement and disease. In addition, most participants felt more confident due to physical therapists’ presence, who took care of them. Most of them also inferred that when they saw daily improvement or felt that they could do more things, they felt encouraged, compared to the starting point.

### Motivation

Most participants told us they felt motivated to perform the exercises proposed by our telerehabilitation programme. A majority related that their motivation increased throughout the programme and they felt better, feeling evolution in the recovery process. Conversely, most of them inferred that they felt more motivated to perform exercises daily since the monitoring performed by the physiotherapists indirectly obligated them to participate with continuity in the telerehabilitation programme.

## 5. Level of Health Assistance

The attention, kindness and flexibility of the physiotherapists that guided the participants during the programme were highlighted. Participants felt safeguarded with being enrolled in the programme. Participants related that the health care provided by the general practitioners in the public health system was deficient during the different pandemic waves and they positively valued the presence of another healthcare provider during the process.

### Applicability to the Public Health System

All of the participants felt it was indispensable and convenient to incorporate telerehabilitation programmes in the health system when managing patients with COVID-19 since they considered it essential to perform follow-ups and subsequently obtain clinical benefit and prevent possible complications and sequelae. In addition, the participants expressed that they felt that such a programme could be extended to other areas. However, some of them thought it was impossible to implement this kind of programme due to the economical or structural issues of the public health system. These participants also expressed consciousness of the current financial problems, reduction in health investments and deficit of healthcare professionals in the public health system that would make it very difficult to implement telerehabilitation programmes, despite their known benefits.

## 6. Discussion

The COVID-19 pandemic has had a massive impact on health care systems worldwide, forcing health professionals to rapidly adapt to the circumstances. During this situation, telerehabilitation has been widely proposed as an exciting way to provide health services and many studies have been published regarding physical therapy implementation during the COVID-19 pandemic [26,27,28]. However, few studies have been documented pertaining to the physical therapy management of COVID-19 patients with telerehabilitation [20]. To our knowledge, this is the first qualitative study describing the opinions and feelings of confined patients with COVID-19 enrolled in a telerehabilitation exercise programme [19]. It has been published that different populations have found the implementation of telerehabilitation programmes to be helpful and they have reported positive experiences with these methods [29,30]. Our results align with these findings in that the participants were generally satisfied with their participation and stated they would participate again. This great acceptability could be related to the fact that patients felt that they took part in their recovery; in that sense, self-efficacy has been very important in managing different conditions [31,32]. Enrolling patients in their treatments is becoming more important nowadays and exercise through telerehabilitation seems an interesting intervention for this purpose. However, to make this achievement possible, new technologies and communication must function correctly. In that sense, our study subjects reflected that no critical problems were found using technologies and contact was qualified as outstanding. Communication is a crucial aspect of health treatments [33,34] and many professionals believe that telerehabilitation could fail in this aspect. However, as it has been shown, this was not the case in the programme developed.

Another critical point of our programme was exercise planning and its adaptability to patients according to perceived effort. In that sense, some subjects pointed out that the programme’s adaptation throughout the 14 days of intervention was beneficial. Adapting the exercise volume according to health status allowed patients in a worse initial state to progress alongside the intervention. There was, however, a negative impact of this action; due to the different clinical pictures in patients with COVID-19 [2], subjects with less COVID-19 severity argued that they would increase the volume/intensity of the exercise programme since it was very easy for them, since the first day. Therefore, individualisation seems crucial for the implementation of these clinical practice interventions, as has been previously argued [35].

In our study, subjects facing the challenge of being part of their recovery and performing exercise for improvement have been described as helping to endure their disease. Despite clinical improvements in respiratory and physical measures, the participants also indicated that they also benefitted from their emotional well-being. We find this point very important since it has been argued in many studies that confinement leads to worsening mental health status in different populations [14,36]. Therefore, our findings highlight that this kind of intervention could help patients managed their disease conditions and prevent worsening mental health. In that sense, many patients have considered that they obtained psychological benefits from this intervention. Emotionally, they highlighted that confinement was not enjoyable and being busy helps to manage this situation. Moreover, the confidence of having a healthcare provider that pays attention to them has been pointed out as very important by many of the participants. Thus, telerehabilitation programmes for COVID-19 patients could improve health status and provide a therapeutic alliance between patients and physical therapists.

In addition, engagement in this programme has increased motivation in patients, which should be an objective of every health intervention. The diary follow-up implemented in this programme contributed to motivating patients to be empowered in managing their diseases.

Patients also described that the feeling of having someone care for them and following their clinical status and evolution was a positive factor in this programme. In their opinion, it was crucial to have the ability to directly ask about any doubt through digital services, if necessary. They argued that public health systems lacked proper follow-up since doctors did not monitor patient evolution in many cases. This point becomes very relevant since health assistance could be improved by using these methods, especially in a confined population, such as COVID-19 patients.

All of the enrolled patients found it indispensable to promote the implementation of telerehabilitation in the public health system, not only for COVID-19 patients, but also for other populations, such as patients with difficulties to displace to patients in health centres, at which effectiveness have already been demonstrated [37,38,39]. Nonetheless, in this situation of uncertainty and reduction of investments in the public health system, it was discovered to be non-viable in the short-term. Increasing the number of physical therapists enrolled in the public system could facilitate the implementation of telerehabilitation methods. However, although desirable, it seems unlikely that this could happen in the short term in the current situation.

Among the limitations, due to the pandemic situation, interviews were conducted by telephone, which may have reduced personal interaction between the participants and researchers; therefore, extracted information could be reduced. Moreover, due to its qualitative design and small sample size, these results cannot be extrapolated to all patients with COVID-19. Readers should consider that this study analyses the experience and feelings of 25% of patients included in our randomised controlled trial. Thus, our conclusions must be taken with care, since although the redundant information extracted from the interviews was achieved, obtaining speeches was very similar. Another issue that we should point out is that we obtained extremely positive results. This finding leads to doubt of the findings themselves. We hypothesise that the subjects’ insecurities due to isolation, disease and fear may have led to very positive feelings about our programme and the perceived attention, avoiding, in a certain way, possible negative aspects about the intervention. However, our findings may assist in the understanding of patients’ feelings regarding their enrolment in a telerehabilitation programme during COVID-19 confinement and their opinions and emotions related to isolation. Moreover, variability in health status due to COVID-19 has been shown to improve our programme. Since patients present symptoms differently, our programme was not demanding for some of them. Therefore, future studies could adapt exercise volume/intensity with more significant variability of series/repetitions.

## 7. Conclusions

The semi-structured interviews performed during this study show that the tele-rehabilitation programme implemented in patients confined by COVID-19 under detailed methods generated significant reception. This result was without considerable technical difficulties in this size study and generated, in the subjects’ opinions, improvements in their physical condition, psychological status, confidence and motivation during the isolation process. The interviewed patients considered it essential to implement this type of assistance in public health systems. Despite these findings, our results should be confirmed with other studies performed with higher sample sizes.

## Figures and Tables

**Table 1:** Participants’ baseline characteristics.

**Age (years)**	41.36 ± 13.36			
**Gender % (*n*)**	Female 76% (18)	Male 24% (7)		
**Civil status % (*n*)**	Married 52% (13)	Single 40% (10)	Divorced 8% (2)	
**Nationality % (*n*)**	Spanish 100% (25)			
**Education % (*n*)**	Secondary 16% (4)	Bachelor 32% (8)	Graduate 16% (4)	Master’s 36% (9)
**Employment situation % (*n*)**	Active 76% (19)	Not active 20% (5)	Retired 4% (1)	

## Data Availability

The data are not publicly available due to privacy and ethicals reasons.

## Appendix A. Interview Questions

- What is your opinion about the telerehabilitation programme in which you were involved?
- How did you feel physically during the telerehabilitation programme?
- How did you feel emotionally during the telerehabilitation programme?
- Did you find impediments to perform the telerehabilitation programme?
- What opinion do you have about the physiotherapeutic approach of your disease through telerehabilitation?

## Appendix B

**Table A1:** Most Highlighted Answers.

Interview Comments
Technical Aspects “*Everything worked perfectly: I could check exercises when I wanted, I performed exercises with the physical therapist that called me… perfect. And I think that it is convenient and easy to be followed. They can call you if any doubts; I think it is good. I looked for them, (laughs) since they always called me at the same hour, I was looking for them.*” “*When the effort test was performed, the APP to count steps did not work; we could not continue this day.*”
Communication “*Sincerely, really good, since the first moment communication worked very well; the health system should go forward in this way.*” “*Fantastic. Displacement was not required, and this was especially important in a sick person. With video calls, everything was straightforward to be understood.*” “*I felt communication was adequate; I was confident since both therapists who contacted me were very nice.*” “*Although it was through video calls, the relationship with therapists was very close and direct.*”
Exercise Planning “*No trouble with exercises. Doubts were immediately solved.*” “*At first, I felt both in the respiratory and physical field that I was a bit limited. Later, I started becoming stronger, and I could perform exercises with less difficulty.*” “*At the beginning, it was like a triathlon. Later, the programme was bearable, and exercises were adapted according to my capacities.*”
Aspects to Improve “*Exercises that I performed were too easy and quiet. They were good for me, but I think that, depending on the grade of severity of COVID-19, exercises volume and intensity should be adapted*.” “*I would increase the duration of the programme since 15 days go very fast when you are in this situation*.” “*Measuring pulsations, instead of how you feel, could become a more objective value, improving the adaptation of exercises*.” “*Maybe the elderly, or other people who do not control new technologies, are at risk of exclusion in this kind of programmes*.” “*Sincerely, I don’t know how the programme could be improved. As I told you, I feel pleased about having participated in the study, can’t find anything to improve. I think that it has been very well prepared since the first movement, and execution, at least in my case, has been excellent*.”
Perception of Clinical Benefit *“Especially the respiratory aspect- excellent… I thought: well, my cough has increased, but I feel like I’m removing and cleaning what I have in there.”* *“Truthfully, I improved m respiratory capacity. I improved since I started doing the exercises because I was vexed…”* “*Truly, I felt my confidence increased, and yes, I’ve felt better. First days, I felt pain in my muscles, and it was tough for me, but later I improved, and I did not feel that way. And, of course, it has influenced my recovery*.” *“One of the most significant symptoms I had, I felt pain in my chest, and I felt charged. For me, it was challenging to go up and down in steps. When I was physically well prepared, I started to sweat the situation, and I felt exhausted. In that situation, performing the exercises undoubtedly helped me, also to understand that I did not have important sequelae, that I could go ahead, and it did not affect me very much.”* “*As I told you, the most important aspect for me was the psychological… Facing a challenge daily, being sure that exercises would help in my recovery and feel better, professionals were guiding me… I was sure that the day after, I was going to feel better. I trusted very much, and then, although I did not find a huge change in the physical aspect, I’m sure that in the psychological aspect, it helped me*.” “*I imagine that I had a clinical benefit, but I don’t know. I felt I had positive benefits in my physical reconditioning*.”
Psychological Aspects *“Well, isolation is not enjoyable. Being busy helped me to get over the process. If you are active, your mood improves.”* “*It has positively influenced me because I knew I had someone who took care of me, a professional with studies and knowledge, and I did not feel alone. It has been conducive for me; confinement implies loneliness, and if there is someone who calls you and your family, it is good, great*.” “… *Yes, sure, psychologically, it helps you because I knew that there was a professional who was there, who was paying attention to me and my situation*.” *“… it was like I was a bit calmer about the follow-up, about the fact that there were some people who are involved in my recovery and how they could help me to improve and get over the disease. Some days I felt alone, but I felt that actually, some people were there to take care of me*.” “*Well, look, it has been very beneficial regarding my mood. I felt with little energy, I felt weakness in my mood, and after performing the exercises, I felt better. This was the main benefit I felt*.”
Motivation “… *I was very unmotivated. I felt generalised weakness, not only back pain but like if I had low energy. Then, it has been beneficial for motivating me to perform exercises and, after doing exercises, I felt better, more encouraged*.” *“…it was like my daily sport because although it was not purely sport, I had not enough strength to do something hard. Therefore, I thought: come, I’m going to perform my respiratory exercises, and I finished my day.”* “*Yes, I saw like I was feeling better, and thus, I felt more motivated. Moreover, indeed, I still perform the exercises sometimes*…” “*Especially in the second or third session, I felt the improvement because the first day I was awful… I could not walk much time, I felt tired, and I could not perform exercises properly, and this situation overwhelmed me. Later, I started to perform exercises better, and I noticed that I was doing faster… Not only that, I felt better, but I was performing the exercises with fewer difficulties*.” “*Depending on the day, there were some days that I felt exhausted, and it was difficult to start, but talking to the therapist indeed gave me confidence and courage to perform exercises.”* “*Yes, some days I felt worse. I did not want to do exercises, but as I know I was going to be contacted to ask me how it had gone, this obligated me to perform the exercises*.”
Level of Health Assistance “*Well, the follow-up, attention, kindness of people who took care of me, and how they adapted to my circumstances… the attention was extraordinary*.” “*Attention has been perfect, and I felt safeguarded. I always had the faculty of asking them in case of any doubt. When I started the programme, I started feeling new things, weird feelings, and having their support in any moment was good*.” “*The direct attention, it is imperative that we had a telephone to consult. Health assistance is very deficient in this situation, and this programme offered us new possibilities*.” “*I had feared to die daily, and they told you that they are going to call you, but… they don’t call you. However, with this programme, I had the security that they were going to contact me. I was pleased about that.”*
Applicability to the public health system “*I think that it should arrive. Not only for patients with COVID-19, but also when COVID-19 disappears, these programmes should stay and they should be developed because, with a good explanation and two video calls, it is sufficient*.” “*It is weird that it does not exist. I do not understand why it is not implemented frequently.”* *“I understand that this is new, that it is not still implemented, but is important and necessary for some patients that could have complications with the facility*.” “*Not only now because of COVID-19, but generally, it should be adopted in the management of patients with mobility problems, difficulties in moving around, people with respiratory issues, in-bed patients… I think it is crucial. Besides, it is not simply a conversation, but it has an objective, a daily programme with a specific aim*.” *“It could be feasible if there were more physiotherapists and they could spend more time with patients.”* *“I think it is not feasible, and it would be complicated. I would like not to say that, but it is complicated. Taking into account that right now is a delicate situation of uncertainty.”* *“I don’t think that the public health system could assume the costs currently. With cuts that the health system is suffering, it would be advisable, but I don’t know if it could be implemented. The public health system does not work well, and I don’t think it would be feasible.”*
